# A Fatal Case of COVID-19 in an Infant with Severe Acute Malnutrition Admitted to a Paediatric Ward in Niger

**DOI:** 10.1155/2020/8847415

**Published:** 2020-09-24

**Authors:** Alido Soumana, Aboubacar Samaila, Lamine Mahaman Moustapha, Moumouni Kamaye, Balkissa Daouda, Ibrahim Alkassoum Salifou, Adamou Lagare, Eric Omar Adehossi, Maman Laminou Ibrahim

**Affiliations:** ^1^Department of Pediatrics, Amirou Boubacar Diallo National Hospital of Niamey, Niamey, Niger; ^2^Sciences of Health Faculty, Abdou Moumouni University of Niamey, Niamey, Niger; ^3^Faculty of Health Science, University of Zinder, Zinder, Niger; ^4^Centre de Recherche Médicale et Sanitaire (CERMES), Niamey, Niger

## Abstract

While there have been very few fatal cases, SARS-CoV-2 has been reported in paediatric patients. This study aims to describe a fatal case of COVID-19 in a child with severe acute malnutrition. The eight-month-old child presented with fever, diarrhoea, and difficulty in breathing. The mother of the child had fever and shortness of breath four weeks before she died. Physical examination revealed lethargy, dehydration, and severe weight loss with a weight of 5 kg at a height of 78 cm tall. The weight-for-height index was less than three *Z*-scores, which corresponds to severe acute malnutrition. The pulmonary examination revealed moderate respiratory distress, and the chest X-ray presented features suggestive of pneumonia in the right lung area. In the context of the COVID-19 outbreak in Niger and the circumstances of the mother's death, a nasal swab was taken for laboratory confirmation. Treatment provided to the child included intranasal oxygen, antibiotics, and a dietary program with therapeutic milk. The child died 48 hours after his admission. The history of contact with a SARS-CoV-2 suspect or positive patient should lead to screening for infection by using RT-PCR. It is important to investigate malnutrition as a potential risk factor for severe SARS-CoV-2 infection and resultant mortality.

## 1. Introduction

The severe acute respiratory syndrome coronavirus 2 (SARS-CoV-2) or coronavirus disease 2019 (COVID-19) is responsible for severe viral pneumonia, which appeared in December 2019 in China [1]. The disease has gradually spread globally. This global health emergency was declared a pandemic by the World Health Organization (WHO) on 30^th^ January 2020 [2].

Few cases of SARS-CoV-2 infection in paediatric patients have been reported compared to adults, limiting clinical data about children [3–6]. During the Wuhan epidemic in China, some cases of children under one year of age have been described [7]. The epidemiological situation of COVID-19 in China has shown that the average age of the patients was 51 years. The morbidity rate varied according to age where patients under 10 years of age represented 1% and those between 10 and 19 years of age represented also 1% of the 72,314 cases in Wuhan. The low number of cases among children and the high frequency of mild clinical symptoms deserve to be investigated [7].

Many risk factors for COVID-19 have been described in the literature, among which older age, asthma, and cardiovascular illnesses contribute significantly [3–5, 8, 9] to the disease. However, the impact of other factors including malnutrition in particular in low-income countries remains unknown.

Therefore, we aim to report a fatal case of COVID-19 in an eight-month-old infant with severe acute malnutrition at the Amirou Boubacar Diallo national hospital (HNABD) of Niamey, Niger.

## 2. Observation

An eight-month-old male child presented with fever, diarrhoea, and difficulty in breathing was admitted to the paediatric ward of the Amirou Boubacar Diallo national hospital (HNABD) of Niamey. The mother of the child had fever and shortness of breath approximately four weeks prior to admission and subsequently died. Upon clinical examination, the child was lethargic with dehydration but without shock or cyanosis, and he weighed 5 kg and was 78 cm in height. The weight-for-height index was less than three *Z*-score, which corresponds to those with severe acute malnutrition. He had tachypnoea with a respiratory rate of 56 cycles/min, tachycardia at 148 heartbeats per minute, and temperature of 38.5°C. Oxygen saturation, measured with a pulse oximeter (SpO_2_), was 90% in ambient air.

The pulmonary examination revealed moderate respiratory distress with chest indrawing. Auscultation showed bronchial rales in the right pulmonary system, and the abdomen was flexible with moderate hepatomegaly.

**Figure 1:**
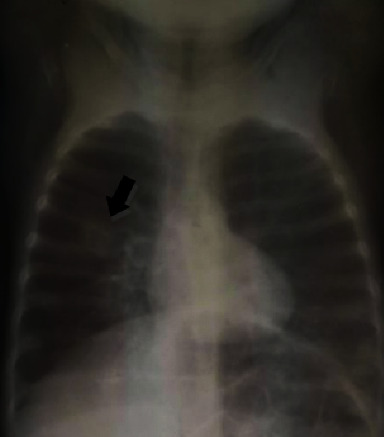
Pneumonia in the right lung.

Based on clinical findings, the epidemic context of COVID-19, and the circumstances of the mother's death, there was a high probability of SARS-CoV-2 infection, as a result of which a nasal swab was taken immediately and sent to the Centre de Recherche Médicale et Sanitaire (CERMES), a National Reference Laboratory for respiratory viruses. Virological analysis was conducted using real-time reverse transcriptase polymerase chain reaction (rRT-PCR). The blood count was normal, the plasma test was negative, and blood glucose was 1.42 g/L. The renal function and blood ion gram were normal. The chest X-ray showed features of pneumonic consolidation in the right lung (Figure 1).

Treatment started immediately with oxygen therapy at 1 l/min, and a combination of ceftriaxone (75 mg/kg/day) and gentamycin (3 mg/kg/day) was instituted. Finally, dietary support was provided with therapeutic milk using a feeding tube. The clinical course was marked by severe respiratory distress and the gradual decrease of SpO_2_, despite oxygen therapy. Unfortunately, the child died 48 hours following admission. The laboratory diagnosis confirmed an infection by SARS-CoV-2 a few hours after the child's death.

## 3. Discussion

This clinical case recounts a fatal SARS-CoV-2 infection in an eight-month-old infant with severe acute malnutrition. To our knowledge, this is the first time that a case of COVID-19 was noticed in an infant with severe acute malnutrition in Niger. SARS-CoV-2 infection was confirmed by RT-PCR by the CERMES of Niamey from nasal swab.

COVID-19 lower respiratory tract infections are rare and often milder among children in general and infants in particular [7]. Therefore, the presence of comorbidity could play an important role in its occurrence [4, 5]. In the study of Shekerdemian et al., the majority of affected children had comorbidities [10]. In this observation, it was a severe acute malnutrition. Malnourished children have an increased susceptibility to infections compared with normally nourished children due to immune system deficiency [11]. Malnutrition is therefore a susceptibility factor for COVID-19 infection. In Niger where malnutrition is common, special attention should be paid to children. Pneumonia is also one of the common infectious complications with these children. However, given the higher risk of clinical severity, monitoring is also recommended. The source of infection for children in most cases is within their family [4–7, 10, 12]. The child's infection could have occurred from his late mother who developed all related symptoms, although laboratory testing was not carried out.

COVID-19 clinical data among children are limited. In the cases reported among infants, the infection was relatively uncomplicated [6]. Symptoms were generally less severe than for adults with variables symptoms like fever, dry cough, and gastrointestinal symptoms like diarrhoea or vomiting [3, 6].

This clinical case initially presented anorexia and diarrhoea for two weeks. Malnutrition may have induced deficiency of the immune system, resulting in the severity and subsequent fatality of SARS-CoV-2 infection. On the radiological level, frosted glass is also the predominant sign in paediatric population. The “halo” sign, corresponding to a nodular opacity surrounded by a crown of frosted glass, is more frequently found with children than adults [3, 12–14].

Case management is difficult in the context of limited personnel and equipment resources. Protection and isolation measures must be scrupulously applied in order to limit the spread of the virus and monitoring is recommended to detect and manage cases. Adequate hydration as well as sufficient calorie intake must be provided [6]. In the absence of an effective vaccine and specific treatment available, it is essential to strengthen COVID-19 diagnosis as early as possible.

This is a first clinical case that documents the comorbidity of SARS-CoV-2 infection with severe acute malnutrition. The limitations to the management of this case were due to the mother's missing COVID-19 infection status.

## 4. Conclusion

SARS-CoV-2 infection is rare in children but presents with a variety of symptoms. The history of contact with a suspected case should lead to a request for laboratory confirmation, especially if there is comorbidity. In the absence of a vaccine and specific treatment, early diagnosis will result in better management of patients and therefore limit the spread of the infection.
